# The joint cardiovascular research profile of the university medical centres in the Netherlands

**DOI:** 10.1007/s12471-016-0828-4

**Published:** 2016-04-04

**Authors:** S. D. van Welie, T. N. van Leeuwen, C. J. Bouma, A. B. M. Klaassen

**Affiliations:** Dutch Heart Foundation, The Hague, the Netherlands; Centre for Science & Technology Studies (CWTS), Leiden, the Netherlands; Netherlands Federation of University Medical Centers, Utrecht, the Netherlands; Dutch Heart Foundation, The Hague, the Netherlands

**Keywords:** Bibliometric analysis, Cardiovascular research topics, International and national competitiveness

## Abstract

Biomedical scientific research in the Netherlands has a good reputation worldwide. Quantitatively, the university medical centres (UMCs) deliver about 40 % of the total number of scientific publications of this research. Analysis of the bibliometric output data of the UMCs shows that their research is highly cited. These output-based analyses also indicate the high impact of cardiovascular scientific research in these centres, illustrating the strength of this research in the Netherlands. A set of six joint national cardiovascular research topics selected by the UMCs can be recognised. At the top are heart failure, rhythm disorder research and atherosclerosis. National collaboration of top scientists in consortia in these three areas is successful in acquiring funding of large-scale programs. Our observations suggest that funding national consortia of experts focused on a few selected research topics may increase the international competitiveness of cardiovascular research in the Netherlands.

## Introduction

The Netherlands has a good reputation worldwide when it comes to biomedical and medical scientific research, most of which takes place in the university medical centres (UMCs). The scientific output of that research can be estimated both quantitatively and qualitatively. Quantitatively, UMCs deliver about 40 % of the total number of scientific publications in the Netherlands [1]. In comparison with other countries, they publish the highest number of scientific articles per million euros of research budget invested [2, http://www.nfu.nl/wetenschap/]. Qualitatively, peer judgments are generally used. Besides that, a suitable indicator of the quality of the output could be derived from bibliometric analysis: the number of times a publication is referred to gives an indication of use by scientists at the research front, as they build on previous work. For the period 2007–2011/2012 the publications of the UMCs were on average cited 56 % more, compared with the world citation average [3].

Cardiovascular scientific research is a branch of this science. Besides a bibliometric analysis of the output of that research, the present study deals with some thematic aspects of cardiovascular scientific research in the UMCs in the Netherlands.

## Background information

On a regular basis the UMCs deliver their annual scientific output to the Centre for Science & Technology Studies (CWTS) and ask this centre to analyse these data on an overall level based on a comparison of the output in journals covered by Thomson Reuters in their Web of Science. Several indicators may be important in a bibliometric analysis as they relate to different aspects of publication and citation characteristics [3]. Generally, the field-normalised impact score, MNCS, is considered to be a powerful internationally standardised impact indicator for analysing research performance [4, 5]. This impact indicator is defined as ‘The impact of a set of research articles, compared with the world citation average in the subfields in which these research papers have appeared’.

## Bibliometric analysis

In this analysis we used the data published in the CWTS report Bibliometric study on Dutch academic medical centres 1998–2011/2012 (http://www.nfu.nl/img/pdf/14.1790_CWTS_Analyse_1998-2011.2012.pdf) [3]. This report is publicly available while the raw data of this report can be derived from the Web of Science. Analysing the bibliometric output data of the UMCs during the period 2007–2011 [3] we create a cardiovascular research profile for each UMC. In that output-based profile two journal subject categories (JSCs) are most characteristic, namely *Cardiac & Cardiovascular Systems* and *Peripheral Vascular Diseases*. These two JSCs are considered to include the most characteristic and specific journals for publishing results of research dealing with the heart and blood vessels. The JSC *Haematology* is not included, because it covers more research that can be related to other types of disease (such as oncology) and to more basic scientific research. However, it is very important to keep in mind that the cardiovascular research profiles only display information on the output and impact per UMC in the two above-mentioned JSCs. You have to realise that these profiles do not provide information on the level of organisational units within a UMC (for instance cardiovascular institutes or departments within a UMC), as there is no direct organic link between the units producing papers and the JSCs these papers belong to [6].

Fig. 9 in the CWTS report [3] displays the research profile for the combined output of the UMCs. The overall MNCS of this output is 1.56 and that of the JSC *Cardiac & Cardiovascular Systems *is 1.64. The MNCS of the JCS *Peripheral Vascular Diseases *is 1.38. In Table 1 the overall MNCS* s*cores in the three categories for the eight UMCs in the Netherlands are presented for the period 2007–2011. All these data indicate the strong position of the medical centres in the international biomedical scientific arena: their research is highly cited. The data for the two relevant cardiovascular categories show a high impact as well, illustrating the strength of the cardiovascular scientific research in the Netherlands.

**Table 1:** Field-normalised impact score (MNCS) in three categories: all journal subject categories (JSCs) combined, JSC cardiac and cardiovascular systems and JSC peripheral vascular diseases for eight UMCs for the period 2007–2011. The total number of fields characteristic for the research profile of each UMC (CWTS report Bibliometric study on Dutch academic medical centers 1998–2011/2012, Figs 1b–8b [3]), is also presented as well as the position of the two relevant cardiovascular JSCs in this total number of fields.

UMC	Field normalised impact score (MNCS)			Research profile of UMC		
	All fields combined	Cardiac & cardiovascular systems	Peripheral vascular diseases	Total fields^a^	Position of the JSC in the total number of fields within the UMC	
					Cardiac & cardiovascular systems	Peripheral vascular diseases
Erasmus MC	1.67	1.61	1.44	28	2	14
LUMC	1.65	1.80	1.40	29	2	13
Radboudumc	1.58	1.50	1.35	33	29	31
Maastricht UMC+	1.55	1.96	1.37	32	2	3
UMC Groningen	1.48	1.38	1.10	31	1	18
UMC Utrecht	1.65	1.68	1.38	29	4	6
AMC	1.56	1.64	1.42	31	1	11
VUMC	1.70	1.77	1.40	32	10	21

^a^In the profiles we only showed those JSCs with over 1 % of the output of the respective UMC

With respect to the JSC *Peripheral Vascular Diseases* we observe MNCS scores of 35 % to 44 % compared with the worldwide average impact level. These scores indicate a highly cited vascular-oriented research field in the Netherlands. Only one UMC has a somewhat lower impact as compared with the other seven UMCs. In the JSC *Cardiac & Cardiovascular Systems* the MNCS scores vary between 1.96 (Maastricht UMC+) and 1.38 (UMC Groningen), indicating that cardiac research in the Netherlands is also highly cited. However, the variations in impact between the eight UMCs are apparently more pronounced in this research field than in the vascular-oriented research field.

In general, between 7.7 % and 9.9 % of the output of a UMC is displayed in its cardiovascular research profile. Exceptions are Radboudumc Nijmegen (2.4 %) and VUMC Amsterdam (4.4 %). Looking at the data of Table 1 we can distinguish several types of cardiovascular research profiles. Maastricht UMC+ has a very high citation score and a strong cardiovascular research profile. Although UMC Utrecht has a lower citation score its cardiovascular research profile may be compared with Maastricht. Three UMCs (Erasmus MC Rotterdam, LUMC Leiden and AMC Amsterdam) have almost comparable cardiovascular research profiles. Radboudumc does not have a strongly marked cardiovascular research profile, but the research in question has a reasonably high citation score. VUMC also has a low cardiovascular research profile, but its cardiovascular research has a high citation score. Compared with the other UMCs, Table 1 also illustrates that UMC Groningen has a somewhat different pattern with an average citation score.

## Cardiovascular research topics

Analysing the bibliometric data of the UMCs, it is interesting to involve the cardiovascular research topics chosen by the respective centres. In April 2014 the websites and the annual reports of the eight UMCs were consulted. In Maastricht cardiovascular research is clustered in the School for Cardiovascular Diseases (CARIM), one of the top institutes for translational cardiovascular research in Europe. In Erasmus MC that research is organised in COEUR and at the VUMC in the Institute for Cardiovascular Research (ICaR-VU). The research program Circulatory Health comprises the cardiovascular research in the UMC Utrecht. In Radboudumc, the Radboud Institute for Molecular Life Sciences (RIMLS) and the Radboud Institute for Health Sciences (RIHS) both distinguish a research theme Vascular Damage. The AMC (Amsterdam) has three cardiovascular key research topics and houses the AMC Heart Failure Research Centre and the Durrer Centre for Cardiogenetic Research. In Groningen the UMC has organised its research in five Research Institutes. One of them (GUIDE) distinguishes the Preservation of Cardiac Function over Time (GUIDE-CVC) program and the Vascular Ageing program (GUIDE-VAP). In Leiden the LUMC has chosen seven medical research profiles, one of which is Vascular and Regenerative Medicine. The Cardiology Department of LUMC mentions two research topics. In Leiden there is also an inter-faculty working group, the Leiden Vascular Medicine, for coronary atherosclerosis. Because the level of the information on cardiovascular research presented by the eight different locations varies substantially it is nearly impossible to give a complete and detailed overview of the cardiovascular research in the Netherlands. At the level of cardiovascular research themes defined as a fair number of connected research projects, a sufficient critical mass of investigators and sufficient output, the results of the analysis are presented in Table 2. This analysis focuses on the cardiovascular diseases specifically mentioned in the information consulted as well as aspects of atherosclerosis.

**Table 2:** Overview of the locations studying cardiovascular diseases as described in the information of the eight university medical centres (status April 2014) in the Netherlands; the locations of atherosclerosis research are also indicated

	University Medical Centre							
	Maastricht UMC+	Erasmus MC	LUMC	VUMC	AMC	UMC Utrecht	UMC Groningen	Radboud umc
**Cardiovascular disease**								
Atherothrombosis/thrombosis	X	X	X		X	X		
Cardiac hypertrophy/ heart failure	X	X	X	X	X	X	X	
Atrial fibrillation	X	X	X				X	
Arrhythmias		X	X		X	X		
Stroke		X			X	X		
Acute coronary syndromes		X	X	X	X		X	
Congenital heart diseases		X	X		X	X	X	
Pulmonary hypertension				X			X	
Heart diseases/vascular aspects and pregnancy				X			X	
Diabetes mellitus	X	X	X	X				
Valvular diseases			X					
**Atherosclerosis**	X	X	X	X	X	X	X	X

Cardiac hypertrophy/heart failure research is a national research topic studied in seven of the eight UMCs. Rhythm disorders are studied in six centres: especially research into ventricular fibrillation in four centres and atrial fibrillation research also in four locations. Atherothrombosis/thrombosis, congenital heart diseases and acute coronary syndromes are topics in five medical centres. The other cardiovascular diseases described in Table 2 are studied in four or less university centres. All UMCs contribute to unravel mechanisms of atherosclerosis and accelerate therapeutic applications in vascular medicine. This analysis clearly accentuates a set of six joint cardiovascular research topics of the eight UMCs.

### International and national competitiveness

Taking into account the cardiovascular research topics, the question that arises is whether top Dutch scientists in each field compete with each other for research funding or collaborate to unravel mechanisms underlying the cardiovascular diseases. And have the investigators already succeeded in establishing multicentre-related research programs? To answer these questions we analysed cardiovascular participation in two large national funding programs and the participation of Dutch cardiovascular scientists as consortium leaders at the European level.

Partly with funding from the natural gas reserve (FES 2006), three Dutch technological institutes were established. One of these institutes, CTMM (Centre for Translational Molecular Medicine) bundles top research groups in universities, knowledge institutes and UMCs with global companies, medium-sized enterprises as well as high tech start-ups, to improve therapies and give a boost to life sciences research and development. The Netherlands CardioVascular Research Initiative (CVON) encourages top scientists to collaborate in national consortia and choose topics that should address the problems encountered in the care of cardiovascular patients [7]. In 2011 and 2013, seven CVON programs were awarded [8, 9]. Consortium leaders at the European level are looked for in the databases of the European Commission for the period 2007–half 2011 (status June 2012).

The results of this analysis are presented in Table 3. This table illustrates the strong position of the two national topics heart failure and rhythm disorders research. In these fields, scientists successfully collaborated and joined forces. In thrombosis research the national collaboration still increases. The remarkable success of research in congenital heart diseases at the European level was apparently due to a few top basic scientists. Our analysis showed that the topic acute coronary syndromes is not yet successful at this level. The strength of Dutch research into vascular and thrombotic disorders is also evident: CTMM CIRCULATING CELLS and CTMM PARISK, CVON GENIUS and CVON IN-CONTROL, Europe EU-PACT (UMC Utrecht) and EU-PHASE (LUMC). The analysis clearly shows that the increasing cooperation of investigators within the top of the selected topics of cardiovascular research in the Netherlands is successful in the international and national competition for funding.

**Table 3:** Multicentre-related cardiovascular research in national funding programs and consortium leadership at the European level

	University Medical Centre							
	Maastricht UMC+	Erasmus MC	LUMC	VUMC	AMC	UMC Utrecht	UMC Groningen	Radboud umc
**Heart failure**								
CTMM TRIUMPH	X	X			X		X	
CVON ARENA	X	X		X	X		X	
Europa MEDIA				X				
Eurostat BIOSTAT-CHG							X	
**Rhythm disorders**								
CTMM COHFAR	X				X	X	X	
CVON PREDICT	X				X	X	X	
**Thrombosis**								
CTMM INCOAG	X		X		X			
**Congenital heart diseases**								
Europa HEART REPAIR					X			
Europa CHEARTED					X			

## Conclusions and summary

Biomedical scientific research in the Netherlands has a worldwide reputation. A branch of this science is the highly cited cardiovascular research of the eight UMCs. A set of six joint national cardiovascular research topics chosen by the UMCs can be recognised. It appears that for the top of these topics (heart failure, rhythm disorders research and atherosclerosis) collaboration of top scientists in national consortia is successful in acquiring funding of large-scale multicentre research programs. These observations may suggest that funding national consortia composed of experts and focused on a few selected research topics addressing the problems encountered in cardiovascular patient care can increase the international profile of Dutch cardiovascular research and its international competitiveness.

### Funding

none

### Conflict of interest

S.D. van Welie, T.N. van Leeuwen, C.J. Bouma and A.B.M. Klaassen state that there are no conflicts of interest.
